# Depression during the COVID-19 pandemic among older Canadians with peptic ulcer disease: Analysis of the Canadian Longitudinal Study on Aging

**DOI:** 10.1371/journal.pone.0289932

**Published:** 2023-10-18

**Authors:** Esme Fuller-Thomson, Hannah Dolhai, Andie MacNeil, Grace Li, Ying Jiang, Margaret De Groh

**Affiliations:** 1 Factor-Inwentash Faculty of Social Work, University of Toronto, Toronto, Ontario, Canada; 2 Institute for Life Course and Aging, University of Toronto, Toronto, Ontario, Canada; 3 Department of Sociology, University of Victoria, Victoria, British Columbia, Canada; 4 Applied Research Division, Center for Surveillance and Applied Research, Public Health Agency of Canada, Ottawa, Ontario, Canada; New York City Department of Health and Mental Hygiene, UNITED STATES

## Abstract

The COVID-19 pandemic and associated public health measures have exacerbated many known risk factors for depression that may be particularly concerning for individuals with chronic health conditions, such as peptic ulcer disease (PUD). In a large longitudinal sample of older adults with PUD, the current study examined the incidence of depression during the pandemic among those without a pre-pandemic history of depression (n = 689) and the recurrence of depression among those with a history of depression (n = 451). Data came from four waves of the Canadian Longitudinal Study on Aging (CLSA). Multivariate logistic regression analyses were conducted to identify factors associated with incident and recurrent depression. Among older adults with PUD and without a history of depression, approximately 1 in 8 (13.0%) developed depression for the first time during the COVID-19 pandemic. Among those with a history of depression, approximately 1 in 2 (46.6%) experienced depression during the pandemic. The risk of incident depression and recurrent depression was higher among those who were lonely, those with functional limitations, and those who experienced an increase in family conflict during the pandemic. The risk of incident depression only was higher among women, individuals whose income did not satisfy their basic needs, those who were themselves ill and/or those whose loved ones were ill or died during the pandemic, and those who had disruptions to healthcare access during the pandemic. The risk of recurrent depression only was higher among those with chronic pain and those who had difficulty accessing medication during the pandemic. Implications for interventions are discussed.

## Introduction

Since the start of the COVID-19 pandemic, there have been more than 6.9 million fatalities worldwide [1], with the United States and Canada experiencing more than 1.1 million and 52,000 deaths, respectively [1, 2]. In response to the pandemic, governments enacted public health measures to mitigate the spread of the virus, such as stay-at-home orders and non-essential business closures. The resulting increases in social isolation and economic precarity, combined with reduced access to health and mental health resources have contributed to declines in physical and mental wellbeing [3, 4] and increases in the prevalence of depression and anxiety in the general population [5, 6].

One particular subpopulation known to have elevated depression prior to the COVID-19 pandemic is individuals with peptic ulcer disease (PUD) [7–9]. PUD is a painful condition in which open gastric sores or ulcers develop in the lining of the stomach or the upper portion of the small intestine. The leading causes of PUD are Helicobacter pylori (H. pylori) infection and long-term use of nonsteroidal anti-inflammatory drugs (NSAIDs), such as aspirin and ibuprofen [10, 11]. Psychological stress is also a major factor contributing to the onset of PUD, as well as the severity of its course [12, 13]. The lifetime prevalence of PUD in the general population is an estimated 5–10% [14]. PUD symptoms, such as bleeding and chronic pain, often disrupt usual activities in daily life, and can result in a dramatically lower quality of life for these individuals [15, 16]. While previous studies have established that depression is associated with an increased risk of developing PUD [8, 9], emerging research suggests that this relationship may be bidirectional, and that those with PUD also face a heightened risk of developing depression [7]. A recent longitudinal study examined the relationship between PUD and depression, and found that individuals with PUD had an adjusted hazard ratio for depression that was 1.68-fold higher than the control group without PUD, even when their demographic factors and other medical history were matched [7].

To date, there has not been a large longitudinal study examining the impact of the COVID-19 pandemic on the mental health of individuals with PUD. While the COVID-19 pandemic exacerbated many risk factors for depression in the general population, there were particular stressors for those with chronic health conditions, who often faced disruptions to their usual healthcare access and increased stress over the risk of COVID-19 [17, 18]. This likely impacted individuals with PUD. Additionally, research conducted prior to the pandemic has attributed the elevated risk of depression among those with PUD to the presence of pain, reduced quality of life, and high levels of stress [7, 19], many of which were likely exacerbated due to the pandemic. Stay-at-home orders and physical distancing limitations had many unintended consequences, such as increases in loneliness and social isolation. It is also possible that the stress of the COVID-19 pandemic led to worsening PUD symptoms, which in turn can lead to worsening mental health outcomes among this population [19]. Collectively, these factors highlight the potentially detrimental impact of the pandemic on the mental health of individuals with PUD.

When considering the high comorbidity between PUD and depression prior to the pandemic, combined with the unprecedented stressors of the COVID-19 pandemic, it is evident that there is a need for longitudinal research to examine the relationship between PUD and the occurrence or recurrence of depression during the pandemic. The current study addresses this gap by using a large Canadian longitudinal panel study of older adults to achieve the following objectives: (1) in a sample of older adults with PUD without a history of depression, to determine the factors associated with developing incident depression during the COVID-19 pandemic; (2) in a sample of older adults with PUD and a history of depression, to identify factors associated with the recurrence of depression during the pandemic.

## Materials and methods

### Data source

As has been described elsewhere [20, 21], data were drawn from the Baseline, Follow-up 1, COVID Spring 2020, and COVID Autumn 2020 waves of the comprehensive cohort of Canadian Longitudinal Study on Aging (CLSA). The CLSA is a national study of Canadian residents aged 45 to 85 between 2011 and 2015 across 10 provinces [22, 23]. Participation in the CLSA cohort is voluntary and all individuals provide written informed consent [22]. The CLSA also collected data about COVID-19, either online or by telephone, with all CLSA respondents who agreed to participate. The Baseline comprehensive cohort recruited 30,097 community-living men and women in Canada. The study was designed to follow participants every 3 years after Baseline for at least 20 years or until death [22]. In total, 27,737 participants of the comprehensive cohort completed the first follow-up wave (hereafter Follow-up 1) in 2018. In order to investigate the impact of the COVID-19 pandemic among older Canadians, 18,530 and 15,544 of the participants in the comprehensive cohort completed the COVID-19 Spring 2020 and Autumn 2020 questionnaires (hereafter Spring 2020 and Autumn 2020). A detailed description of the CLSA can be found elsewhere [22, 23].

All Canadian Longitudinal Study on Aging (CLSA) waves of data collection have been approved by research ethics boards at all collaborating Canadian institutions. The CLSA was conducted in accordance with the 1964 Helsinki declaration and its later amendments, and with the ethical standards of each institutional research committee. The current study was approved by the University of Toronto’s Research Ethics Board (Protocol #41167; approved June 4, 2021).

### Sample

The sample consisted of respondents who had PUD in either Baseline or Follow-up 1 prior to the COVID-19 pandemic. Self-reported PUD was measured by the question, “Has a doctor ever told you that you have intestinal or stomach ulcers?” (yes vs. no). There were 1,293 individuals who reported having PUD. Out of these individuals, 11.8% (n = 153) had missing data on at least one of the covariates and were removed from the analysis. This resulted in a final sample size of 1,140. The variable with the highest percentage of missing was adverse childhood experiences (ACEs), with 2.6% of respondents (n = 33) missing data. The demographic characteristics of respondents who were excluded from the analyses due to missing data were compared to those included in the analyses, and the results showed that they were comparable. Additionally, the missing values appeared to be random and lacked any discernible pattern. Among the final sample of those who reported having PUD and who had data on all covariates (n = 1,140), 689 had no pre-pandemic history of depression and 451 had a history of depression.

### Measures

The outcome of interest, depression, was assessed by administering the short form of the Centre for Epidemiologic Studies–Depression (CES-D-10) scale [24]. Ten questions addressing, for example, feelings of depression, loneliness, hopefulness for the future, and restless sleep, are summed to generate a total score between 0 and 30 with higher scores indicating a greater number of symptoms. The positive screen for depression was coded 1 if the CES-D-10 score was 10 or more and 0 otherwise [24].

To identify pre-pandemic history of depression, four measures were used. The CES-D-10 scores at Baseline and Follow-up 1 wave of data collection were assessed. At both Baseline and Follow-up 1 the respondents were also asked: “Has a doctor ever told you that you suffer from clinical depression?”. If respondents answered no at both the Baseline and Follow-up 1 waves of data collection, and screened negative on the CES-D-10 in both waves, the respondent was classified as having no pre-pandemic history of depression. If at least one of the above four measures indicated depression, the respondent was classified as having a pre-pandemic history of depression.

Demographic factors included age at the Autumn 2020 wave, sex (male vs. female), and marital status (married/common-law, separated/divorced/widowed, single/never married or lived with a partner). Immigrant status was categorized into two groups based on country of origin (Canadian-born vs. immigrant). Visible minority status was classified as white vs. non-white.

Socioeconomic status was measured by education, household income, house ownership, total savings, and whether income satisfies basic needs. We operationalized education as the highest level of education achieved, categorized as less than secondary school, secondary school/some post-secondary, and post-secondary degree/diploma. Household income was defined as total household income from all sources by all family members in the past 12 months (less than $50,000, $50,000–$99,999, $100,000 or more, and not answered). House ownership had three categories (rent, own with mortgage, own without mortgage). Total saving had four categories (less than $50,000, $50,000–$99,999, $100,000 or more, and not answered). Whether income satisfies basic needs was measured by a dummy variable (0 = with some difficulty/not very well/totally inadequate; and 1 = very well/adequately).

Health-related variables include body mass index (BMI), chronic pain, and multimorbidity. BMI was classified into three clusters: underweight/normal weight (BMI < 25.0); overweight (BMI = 25.0–29.9); and obese (BMI ≥ 30.0). Chronic pain was a dummy variable (free of chronic pain vs. have chronic pain). Multimorbidity was defined as having multiple chronic conditions (zero, one, two, three or more, missing). These chronic conditions include (1) diabetes, (2) heart disease, (3) peripheral vascular disease or poor circulation in limbs, (4) dementia or Alzheimer’s disease, (5) multiple sclerosis, (6) epilepsy, (7) migraine headaches, (8) chronic obstructive pulmonary disease or asthma, (9) bowel disorder, (10) stroke or CVA (cerebrovascular accident), (11) glaucoma, (12) kidney disease, (13) macular degeneration, (14) ministroke or TIA (Transient Ischemic Attack), and (15) Parkinson’s Disease, and (16) cancer.

Adverse childhood experiences (ACEs) were measured by childhood physical abuse, childhood sexual abuse, childhood exposure to intimate partner violence, and being neglected. Childhood physical abuse was present if the respondent reported being kicked, bit, punched, choked, burned. or physically attacked in some other way one or more times. Childhood sexual abuse was present if respondents reported an adult forcing them or attempting to force them into any unwanted sexual activity by threatening them, holding them down, or hurting them in some way one or more times. Childhood exposure to intimate partner violence was present if respondents reported seeing or hearing parents, step-parents or guardians hitting each other more than 10 times. Respondents being neglected were present if they reported their parents or guardians not having taken care of their basic needs such as keeping clean or providing food or clothing one or more times.

Respondents were also asked: “How often do you feel that you lack companionship?”, “How often do you feel left out?”, and “How often do you feel isolated from others?”. The three variables were coded as 1 = often and 0 = hardly ever/some of the time.

Religiosity was measured by two questions: “In the past 12 months, how often did you participate in church or religious activities?” and “In the past 12 months, how often did you engage in religious or spiritual activities including prayer, meditation taking place at home?”. If respondents had taken part in these activities at least once a month, then they were coded as often (= 1) otherwise rare (= 0).

At the beginning of the COVID-19 pandemic, the Spring 2020 questionnaire asked respondents whether they left home or not in the past month. Moreover, respondents’ loneliness during the Spring 2020 wave was operationalized using the question, “How often did you feel lonely?” (rarely or never/some of the time [0–2 days per week] vs. occasionally/all of the time [3–7 days per week]). The type of dwelling during the COVID-19 pandemic was classified as house, apartment, or other. There was a dummy variable indicating whether respondents lived alone or not during the beginning of the COVID-19 pandemic.

The functional limitation scale included three questions: “Do you have any difficulty standing up after sitting in a chair”, “Do you have any difficulty walking alone up and down a flight of stairs?”, and “Do you have any difficulty walking 2 to 3 neighborhood blocks?”. If respondents answered all three questions as no, then they were coded as 0, otherwise 1.

COVID-19-related stressors were measured at the Autumn 2020 wave. They were investigated in a section of the survey which was preceded by the following instructions: “Which of the following have you experienced during the COVID-19 pandemic?” COVID-19 related stressors/experiences were categorized into five composite indicators. Experiences were grouped as “yes” if the participants responded yes to at least one experience in the specific category or no if the participant responded no to all questions in that category. Questions related to health stressors included, “You were ill”, “People close to you were ill”, and/or “Death of a person close to you”. Questions related to difficulties with accessing resources included “Loss of income” and/or “Unable to access necessary supplies or food”. Questions related to family conflict included “Increased verbal or physical conflict” and/or “Breakdown in family/marital relationships”. Questions related to other family issues included “Separation from family”, “Increased time caregiving”, and/or “Unable to care for people who require assistance due to health condition or limitation”. Questions related to health care included “Unable to access my usual healthcare”. Questions related to medication included “Unable to get my usual prescription medications and treatments”.

### Analyses

The analysis was conducted in several steps. The variables included in the analyses were determined a priori based upon the existing literature on risk factors associated with depression among older adults [25]. First, descriptive statistics were used to characterize the CLSA participants with PUD, both with and without a pre-pandemic history of depression. We further compared the distribution of key risk factors among older adults with PUD and no pre-pandemic history of depression who did not develop depression and those who develop depression, and older adults with PUD and a history of pre-pandemic depression who did not and those who did develop depression during the pandemic. In order to compare the pandemic incident and recurrent depression rates to pre-pandemic rates, we conducted a sensitivity analysis examining changes in depression from Baseline to Follow-up 1. Chi-square tests and independent t-tests were used to test the statistical differences at the bivariate level. Next, multivariable logistic regression was utilized to analyze incident depression among older adults with PUD who had no prior history of depression. In contrast, relative risk regression was employed to examine the relationship between depression and older adults with PUD who had a pre-existing history of depression. When the outcome of interest is rare, logistic regression is commonly used to estimate the odds ratio, which provides an approximation of the relative risk. However, when the outcome is not rare, a relative risk model is more appropriate as it directly estimates the relative risk, offering a more accurate measure of the association between the predictors and the outcome [26]. The dependent variable is based on the presence or absence of a positive screen for depression based on the CES-D-10 scores at the Autumn 2020 survey. All hypothesis tests were two-sided and p-values less than 0.05 were considered statistically significant. In order to evaluate the goodness-of-fit of logistic models, Nagelkerke R square was reported. We calculated the variance inflation factor (VIF) to assess the multicollinearity among the independent variables in the logistic regression. The VIF values were all below 5, which suggests multicollinearity is not problematic. We also calculated the VIF to assess multicollinearity among the socioeconomic status variables, and we found no evidence for multicollinearity as all VIF values were below 5. Data manipulation and statistical analyses were performed using R version 4.1.3.

## Results

Table 1 presents the sample characteristics of individuals with PUD by pre-pandemic depression status (estimated using CES-D-10 score and self-reported clinical depression diagnosis). A larger proportion of female respondents had a pre-pandemic history of depression than male respondents. A higher proportion of separated/divorced/widowed respondents and single respondents had a pre-pandemic history of depression than those who were married or living common-law. Sociodemographic and health-related variables were different between those who were free of pre-pandemic depression and those who had a history of depression. Respondents who had no history of pre-pandemic depression had a higher household income, owned their house without mortgage, had more savings, had enough income to satisfy needs, were under or normal weight, free of chronic pain, and had no multimorbidity when compared to those who had a history of pre-pandemic depression. A higher proportion of respondents who had a history of pre-pandemic depression felt a lack of companionship, felt left out, felt isolated from others, felt lonely during the pandemic, had functional limitations, and experienced COVID-related stressors.

**Table 1:** Characteristics of CLSA respondents with PUD (n = 1,140).

	Overall sample of older adults with PUD (n = 1,140)	Older adults with PUD with no pre-pandemic history of depression (n = 689)	Older adults with PUD with a pre-pandemic history of depression (n = 451)	p-value	Source of data
Depression during Autumn 2020					CLSA Autumn 2020
No	842 (73.9%)	600 (87.1%)	242 (53.7%)		
Yes	298 (26.1%)	89 (12.9%)	209 (46.3%)		
Age (Mean, SD)	64.06 (9.07)	64.84 (8.94)	62.86 (9.15)	<0.001	CLSA Autumn 2020
Sex				<0.001	CLSA Baseline
Female	598 (52.5%)	315 (45.7%)	283 (62.7%)		
Male	542 (47.5%)	374 (54.3%)	168 (37.3%)		
Marital status				<0.001	CLSA Follow-up 1
Married/Common-law	758 (66.5%)	492 (71.4%)	266 (59.0%)		
Separated/Divorced/Widowed	276 (24.2%)	147 (21.3%)	129 (28.6%)		
Single	106 (9.3%)	50 (7.3%)	56 (12.4%)		
Immigrant status				0.025	CLSA Baseline
No	947 (83.1%)	558 (81.0%)	389 (86.3%)		
Yes	193 (16.9%)	131 (19.0%)	62 (13.7%)		
Visible minority status				0.893	CLSA Baseline
No	1,087 (95.4%)	656 (95.2%)	431 (95.6%)		
Yes	53 (4.6%)	33 (4.8%)	20 (4.4%)		
Education				0.253	CLSA Baseline
Less than secondary school	79 (6.9%)	43 (6.2%)	36 (8.0%)		
Some post-secondary school	203 (17.8%)	116 (16.8%)	87 (19.3%)		
Post-secondary degree/diploma	858 (75.3%)	530 (76.9%)	328 (72.7%)		
Household income				<0.001	CLSA Follow-up 1
Less than $50,000	294 (25.8%)	139 (20.2%)	155 (34.4%)		
$50,000–$99,999	441 (38.7%)	277 (40.2%)	164 (36.4%)		
$100,000 or more	343 (30.1%)	229 (33.2%)	114 (25.3%)		
Missing	62 (5.4%)	44 (6.4%)	18 (4.0%)		
House ownership				<0.001	CLSA Follow-up 1
Rent	185 (16.2%)	76 (11.0%)	109 (24.2%)		
Own with mortgage	313 (27.5%)	175 (25.4%)	138 (30.6%)		
Own without mortgage	622 (54.6%)	426 (61.8%)	196 (43.5%)		
Missing	20 (1.8%)	12 (1.7%)	8 (1.8%)		
Total saving				<0.001	CLSA Follow-up 1
Less than $49,999	247 (21.7%)	110 (16.0%)	137 (30.4%)		
$50,000–$99,999	150 (13.2%)	83 (12.0%)	67 (14.9%)		
$100,000 or more	655 (57.5%)	439 (63.7%)	216 (47.9%)		
Missing	88 (7.7%)	57 (8.3%)	31 (6.9%)		
Whether income satisfies needs				<0.001	CLSA Baseline
No	111 (9.7%)	38 (5.5%)	73 (16.2%)		
Yes	1,029 (90.3%)	651 (94.5%)	378 (83.8%)		
BMI				<0.001	CLSA Follow-up 1
Underweight or normal weight	288 (25.3%)	187 (27.1%)	101 (22.4%)		
Overweight	466 (40.9%)	307 (44.6%)	159 (35.3%)		
Obese	386 (33.9%)	195 (28.3%)	191 (42.4%)		
Chronic pain				<0.001	CLSA Follow-up 1
No	652 (57.2%)	451 (65.5%)	201 (44.6%)		
Yes	488 (42.8%)	238 (34.5%)	250 (55.4%)		
Multimorbidity				<0.001	CLSA Follow-up 1
0	235 (20.6%)	169 (24.5%)	66 (14.6%)		
1	314 (27.5%)	210 (30.5%)	104 (23.1%)		
2	259 (22.7%)	146 (21.2%)	113 (25.1%)		
3+	289 (25.4%)	138 (20.0%)	151 (33.5%)		
Missing	43 (3.8%)	26 (3.8%)	17 (3.8%)		
Feel that lack companionship				<0.001	CLSA Follow-up 1
No	1,059 (92.9%)	670 (97.2%)	389 (86.3%)		
Yes	81 (7.1%)	19 (2.8%)	62 (13.7%)		
Feel left out				<0.001	CLSA Follow-up 1
No	1,098 (96.3%)	680 (98.7%)	418 (92.7%)		
Yes	42 (3.7%)	9 (1.3%)	33 (7.3%)		
Feel isolated from others				<0.001	CLSA Follow-up 1
No	1,104 (96.8%)	686 (99.6%)	418 (92.7%)		
Yes	----	----	----		
Church or religious activities				<0.001	CLSA Follow-up 1
Rarely	767 (67.3%)	435 (63.1%)	332 (73.6%)		
Often	373 (32.7%)	254 (36.9%)	119 (26.4%)		
Religious activities at home				0.870	CLSA Follow-up 1
Rarely	511 (44.8%)	307 (44.6%)	204 (45.2%)		
Often	629 (55.2%)	382 (55.4%)	247 (54.8%)		
Adverse childhood experience (Mean, SD)	0.31 (0.70)	0.20 (0.56)	0.48 (0.84)	<0.001	CLSA Follow-up 1
Left home in the past one month during COVID				0.361	CLSA Spring 2020
No	92 (8.1%)	51 (7.4%)	41 (9.1%)		
Yes	1,048 (91.9%)	638 (92.6%)	410 (90.9%)		
How often do you feel lonely during COVID				<0.001	CLSA Spring 2020
Rarely or never/Some of the time	940 (82.5%)	620 (90.0%)	320 (71.0%)		
Occasionally/All of the time	200 (17.5%)	69 (10.0%)	131 (29.0%)		
Type of dwelling				<0.001	CLSA Spring 2020
House	835 (73.2%)	532 (77.2%)	303 (67.2%)		
Apartment	258 (22.6%)	144 (20.9%)	114 (25.3%)		
Others	47 (4.1%)	13 (1.9%)	34 (7.5%)		
Living along during the COVID-19 pandemic				<0.001	CLSA Spring 2020
No	825 (72.4%)	529 (76.8%)	296 (65.6%)		
Yes	315 (27.6%)	160 (23.2%)	155 (34.4%)		
Functional limitation				<0.001	CLSA Autumn 2020
No	786 (68.9%)	512 (74.3%)	274 (60.8%)		
Yes	354 (31.1%)	177 (25.7%)	177 (39.2%)		
COVID _ Health stressors				0.001	CLSA Autumn 2020
No	718 (63.0%)	462 (67.1%)	256 (56.8%)		
Yes	422 (37.0%)	227 (32.9%)	195 (43.2%)		
COVID _ Difficulties with accessing resources				<0.001	CLSA Autumn 2020
No	948 (83.2%)	595 (86.4%)	353 (78.3%)		
Yes	192 (16.8%)	94 (13.6%)	98 (21.7%)		
COVID _ Family conflict				<0.001	CLSA Autumn 2020
No	1,009 (88.5%)	635 (92.2%)	374 (82.9%)		
Yes	131 (11.5%)	54 (7.8%)	77 (17.1%)		
COVID _ Other family Issues				0.008	CLSA Autumn 2020
No	488 (42.8%)	317 (46.0%)	171 (37.9%)		
Yes	652 (57.2%)	372 (54.0%)	280 (62.1%)		
COVID _ Health care				0.004	CLSA Autumn 2020
No	795 (69.7%)	503 (73.0%)	292 (64.7%)		
Yes	345 (30.3%)	186 (27.0%)	159 (35.3%)		
COVID _ Medications				0.008	CLSA Autumn 2020
No	1,054 (92.5%)	649 (94.2%)	405 (89.8%)		
Yes	86 (7.5%)	40 (5.8%)	46 (10.2%)		

Note: ----indicates a value that could not be estimated due to insufficient cell size.

Table 2 displays the characteristics of older adults with PUD with and without a history of depression who did not develop depression and who developed depression. Among older adults with PUD and no pre-pandemic history of depression, 13.0% developed depression during the pandemic. Among older adults with PUD with a history of depression, nearly half (46.6%) were depressed during the pandemic. More than 7 in 10 older adults with PUD (71.2%) who screened positive for depression based on CES-D-10 scores at both the Baseline and Follow-up 1 waves were depressed in the Autumn 2020 wave, compared to half of those (50.0%) who screened positive for depression based on CES-D-10 scores at Follow-up 1 wave, but not at Baseline, and 44.1% of those whose CES-D-10 scores indicated depression at Baseline, but not at Follow-up 1. In contrast, 29.0% of those who screened negative for depression on the CES-D-10 scores at both Baseline and Follow-up 1 waves, but who reported that they had been diagnosed by a health professional at some point in their life, developed depression during the pandemic.

**Table 2:** Cumulative incidence of depression by CLSA Autumn 2020 among older adults with PUD.

	Older adults with PUD with no history of depression who did not develop depression (n = 600)	Older adults with PUD with no history of depression who developed depression (n = 89)	p-value	Older adults with PUD with a history of depression who did not experience a recurrence of depression (n = 242)	Older adults with PUD with a history of depression who experienced a recurrence of depression (n = 209)	p-value
History of depression prior to pandemic			<0.001			<0.001
No history of depression	600 (87.1%)	89 (12.9%)		-	-	
Any history of depression				242 (53.7%)	209 (46.3%)	
Reported diagnosed by a health professional but not depressed at Baseline or Follow-up 1	-	-		110 (71.0%)	45 (29.0%)	
Depressed at Baseline	-	-		57 (55.9%)	45 (44.1%)	
Depressed at Follow-up 1	-	-		45 (50.0%)	45 (50.0%)	
Depressed at Baseline and Follow-up 1	-	-		30 (28.8%)	74 (71.2%)	
Age	64.92 (8.95)	64.31 (8.89)	0.550	63.15 (8.97)	62.52 (9.36)	0.465
Sex			0.002			0.945
Male	340 (90.9%)	34 (9.1%)		91 (54.2%)	77 (45.8%)	
Female	260 (82.5%)	55 (17.5%)		151 (53.4%)	132 (46.6%)	
Marital status			0.740			0.020
Married/Common-law	431(87.6%)	61 (12.4%)		155 (58.3%)	111 (41.7%)	
Separated/Divorced/Widowed	127 (86.4%)	20 (13.6%)		56 (43.4%)	73 (56.6%)	
Single	42 (84.0%)	8 (16.0%)		31 (55.4%)	25 (44.6%)	
Immigrant status			0.117			0.628
No	480 (86.0%)	78 (14.0%)		211 (54.2%)	178 (45.8%)	
Yes	120 (91.6%)	11 (8.4%)		31 (50.0%)	31 (50.0%)	
Visible minority status			1.000			1.000
No	571 (87.0%)	85 (13.0%)		231 (53.6%)	200 (46.4%)	
Yes	----	----		11 (55.0%)	9 (45.0%)	
Education			0.291			0.566
Less than secondary school	37 (86.0%)	6 (14.0%)		22 (61.1%)	14 (38.9%)	
Secondary and some post-secondary	96 (82.8%)	20 (17.2%)		44 (50.6%)	43 (49.4%)	
Post-secondary degree/diploma	467 (88.1%)	63 (11.9%)		176 (53.7%)	152 (46.3%)	
Household income			0.240			0.320
Less than $50,000	117 (84.2%)	22 (15.8%)		80 (51.6%)	75 (48.4%)	
$50,000–$99,999	239 (86.3%)	38 (13.7%)		87 (53.0%)	77 (47.0%)	
$100,000 or more	202 (88.2%)	27 (11.8%)		68 (59.6%)	46 (40.4%)	
Missing	----	----		7 (38.9%)	11 (61.1%)	
House ownership			0.165			0.440
Rent	69 (90.8%)	7 (9.2%)		55 (50.5%)	54 (49.5%)	
Own with mortgage	158 (90.3%)	17 (9.7%)		69 (50.0%)	69 (50.05%)	
Own without mortgage	364 (85.4%)	62 (14.6%)		113 (57.7%)	83 (42.3%)	
Missing	----	----		----	----	
Total saving			0.914			0.087
Less than $49,999	97 (88.2%)	13 (11.8%)		62 (45.3%)	75 (54.7%)	
$50,000–$99,999	72 (86.7%)	11 (13.3%)		35 (52.2%)	32 (47.8%)	
$100,000 or more	380 (86.6%)	59 (13.4%)		126 (58.3%)	90 (41.7%)	
Missing	51 (89.5%)	6 (10.5%)		19 (61.3%)	12 (38.7%)	
Whether income satisfies needs			0.197			<0.001
No	30 (78.9%)	8 (21.1%)		25 (34.2%)	48 (65.8%)	
Yes	570 (87.6%)	81 (12.4%)		217 (57.4%)	161 (42.6%)	
BMI			0.826			0.769
Underweight or normal weight	165 (88.2%)	22 (11.8%)		51 (50.5%)	50 (49.5%)	
Overweight	265 (86.3%)	42 (13.7%)		87 (54.7%)	72 (45.3%)	
Obese	170 (87.2%)	25 (12.8%)		104 (54.5%)	87 (45.5%)	
Chronic pain			0.857			<0.001
No	394 (87.4%)	57 (12.6%)		130 (64.7%)	71 (35.3%)	
Yes	206 (86.6%)	32 (13.4%)		112 (44.8%)	138 (55.2%)	
Multimorbidity			0.946			0.188
0	150 (88.8%)	19 (11.2%)		38 (57.6%)	28 (42.4%)	
1	180 (85.7%)	30 (14.3%)		65 (62.5%)	39 (37.5%)	
2	126 (86.3%)	20 (13.7%)		60 (53.1%)	53 (46.9%)	
3+	121 (87.7%)	17 (12.3%)		71 (47.0%)	80 (53.0%)	
Missing	----	----		8 (47.1%)	9 (52.9%)	
Feel they lack companionship			0.005			0.007
No	588 (87.8%)	82 (12.2%)		219 (56.3%)	170 (43.7%)	
Yes	12 (63.2%)	7 (36.8%)		23 (37.1%)	39 (62.9%)	
Feel left out			0.019			0.127
No	595 (87.5%)	85 (12.5%)		229 (54.8%)	189 (45.2%)	
Yes	----	-----		13 (39.4%)	20 (60.6%)	
Feel isolated from others			0.846			0.009
No	598 (87.2%)	88 (12.8%)		232 (55.5%)	186 (44.5%)	
Yes	----	----		10 (30.3%)	23 (69.7%)	
Church or religious activities			0.180			0.002
Rarely	385 (88.5%)	50 (11.5%)		163 (49.1%)	169 (50.9%)	
Often	215 (84.6%)	39 (15.4%)		79 (66.4%)	40 (33.6%)	
Religious activities at home			0.011			0.038
Rarely	279 (90.9%)	28 (9.1%)		98 (48.0%)	106 (52.0%)	
Often	321 (84.0%)	61 (16.0%)		144 (58.3%)	103 (41.7%)	
Adverse childhood experience	0.18 (0.52)	0.31 (0.76)	0.040	0.40 (0.76)	0.56 (0.92)	0.039
Left home in the past one month during COVID			0.970			0.250
No	45 (88.2%)	6 (11.8%)		18 (43.9%)	23 (56.1%)	
Yes	555 (87.0%)	83 (13.0%)		224 (54.6%)	186 (45.4%)	
How often do you feel lonely during COVID			<0.001			<0.001
Rarely or never/Some of the time	556 (89.7%)	64 (10.3%)		194 (60.6%)	126 (39.4%)	
Occasionally/All of the time	44 (63.8%)	25 (36.2%)		48 (36.6%)	83 (63.4%)	
Type of dwelling			0.095			0.643
House	471 (88.5%)	61 (11.5%)		162 (53.5%)	141 (46.5%)	
Apartment	119 (82.6%)	25 (17.4%)		64 (56.1%)	50 (43.9%)	
Others	----	----		16 (47.1%)	18 (52.9%)	
Living along during the COVID-19 pandemic			0.194			0.034
No	466 (88.1%)	63 (11.9%)		170 (57.4%)	126 (42.6%)	
Yes	134 (83.8%)	26 (16.3%)		72 (46.5%)	83 (53.5%)	
Functional limitation			0.006			<0.001
No	457 (89.3%)	55 (10.7%)		171 (62.4%)	103 (37.6%)	
Yes	143 (80.8%)	34 (19.2%)		71 (40.1%)	106 (59.95%)	
COVID _ Health stressors			<0.001			0.034
No	424 (91.8%)	38 (8.2%)		149 (58.2%)	107 (41.8%)	
Yes	176 (77.5%)	51 (22.5%)		93 (47.7%)	102 (52.3%)	
COVID _ Difficulties with accessing resources			0.015			0.105
No	526 (88.4%)	69 (11.6%)		197 (55.8%)	156 (44.2%)	
Yes	74 (78.7%)	20 (21.3%)		45 (45.9%)	53 (54.1%)	
COVID _ Family conflict			<0.001			<0.001
No	562 (14.7%)	73 (11.5%)		222 (59.4%)	152 (40.6%)	
Yes	38 (70.4%)	16 (29.6%)		20 (26.0%)	57 (74.0%)	
COVID _ Other family Issues			<0.001			0.037
No	292 (92.1%)	25 (7.9%)		103 (60.2%)	68 (39.8%)	
Yes	308 (82.8%)	64 (17.2%)		139 (49.6%)	141 (50.4%)	
COVID _ Health care			0.001			<0.001
No	452 (89.9%)	51 (10.1%)		176 (60.3%)	116 (39.7%)	
Yes	148 (79.6%)	38 (20.4%)		66 (41.5%)	93 (58.5%)	
COVID _ Medications			0.105			<0.001
No	569 (87.7%)	80 (12.3%)		234 (57.8%)	171 (42.2%)	
Yes	31 (77.5%)	9 (22.5%)		8 (17.4%)	38 (82.6%)	

Note: ----indicates that a value could not be estimated due to insufficient cell size.

We conducted a sensitivity analysis to compare these findings on incident and recurrent depression among older adults with PUD during the COVID-19 pandemic to pre-pandemic patterns from the Baseline to the Follow-up 1 wave. Among those who reported they had no history of depression and a CES-D-10 score less than 10 at Baseline, the incidence of new depression at Follow-up 1 according to the CES-D-10 was 7.5% (95% CI: 5.4%, 9.6%). Among those who had a CES-D-10 score of 10 or more and/or a self-reported history of a medical diagnosis of depression at Baseline, the prevalence of depression at Follow-up 1 was 37.9% (95% CI: 32.4%, 43.5%). As reported above, the COVID-19 incidence and recurrence rates of depression were substantially higher than pre-pandemic rates.

Table 3 presents the multivariate logistic regression model on incident depression status during Autumn 2020 for older adults with PUD and no history of depression. Female respondents had substantially higher odds of incident depression than male respondents during the COVID-19 pandemic (OR = 2.03, 95% CI [1.13; 3.69], p = 0.021). Older adults with PUD who felt lonely occasionally/all of the time during the first few months of the pandemic had a significantly higher prevalence of depression by Autumn 2020 compared to those who felt lonely rarely or never/some of the time (OR = 5.99, 95% CI [2.87, 12.48], p < 0.001). Respondents living in apartments were more likely to report depression during the pandemic than those who lived in houses (OR = 2.55, 95% CI [1.24; 5.24], p = 0.011). Respondents with functional limitations also reported higher odds of incident depression compared to those without (OR = 2.69, 95% CI [1.43; 5.05], p = 0.002). Experiencing COVID-19-related health stressors, such as becoming ill or having a loved one become ill or die, was significantly associated with higher odds ratio of developing incident depression (OR = 3.72, 95% CI [2.12; 6.55], p <0.001). Moreover, older adults with PUD who had increased verbal or physical conflict in the family during the COVID-19 pandemic had a higher odds ratio of developing incident depression (OR = 3.30, 95% CI [1.47; 7.43], p = 0.004). Respondents who were unable to access usual healthcare had higher odds for depression (OR = 1.91, 95% CI [1.04; 3.50], p = 0.037) compared to those who were able to access usual health care.

**Table 3:** Logistic regression results for incident depression among older adults with PUD with no history of depression (n = 689).

	Adjusted Odds Ratio	95% Confidence Interval	p-value	
Age	0.98	[0.95; 1.02]		0.370
Sex				
Male (ref.)				
Female	2.03*	[1.13; 3.69]		0.021
Marital status				
Married/Common-law (ref.)				
Separated/Divorced/Widowed	0.63	[0.24; 1.67]		0.353
Single	1.49	[0.43; 5.21]		0.531
Immigrant status				
No (ref.)				
Yes	0.47	[0.21; 1.06]		0.068
Visible minority status				
No (ref.)				
Yes	1.94	[0.51; 7.38]		0.330
Education				
Less than secondary school	1.55	[0.45; 5.34]		0.487
Secondary and some post-secondary	1.71	[0.84; 3.49]		0.142
Post-secondary degree/diploma (ref.)				
Household income				
Less than $50,000 (ref.)				
$50,000–$99,999	1.89	[0.78; 4.56]		0.159
$100,000 or more	1.57	[0.57; 4.30]		0.381
Missing	0.17	[0.03; 1.07]		0.058
Dwelling ownership				
Rent (ref.)				
Own with mortgage	1.21	[0.35; 4.16]		0.760
Own without mortgage	2.84	[0.91; 8.89]		0.072
Missing	4.59	[0.59;35.56]		0.145
Total saving				
Less than $49,999 (ref.)				
$50,000–$99,999	3.31*	[1.07; 10.26]		0.038
$100,000 or more	2.10	[0.80; 5.51]		0.133
Missing	1.89	[0.49; 7.33]		0.360
Whether income satisfies needs				
No	3.67*	[1.14; 11.82]		0.030
Yes (ref.)				
BMI				
Underweight or normal weight (ref.)				
Overweight	1.34	[0.69; 2.63]		0.386
Obese	0.69	[0.32; 1.49]		0.341
Chronic pain				
No (ref.)				
Yes	0.65	[0.35; 1.18]		0.157
Multimorbidity				
0 (ref.)				
1	1.30	[0.62; 2.70]		0.486
2	1.05	[0.45; 2.43]		0.917
3+	0.87	[0.37; 2.07]		0.753
Missing	1.44	[0.33; 6.25]		0.630
Feel they lack companionship				
No (ref.)				
Yes	4.22	[0.96; 18.61]		0.057
Feel left out				
No (ref.)				
Yes	2.74	[0.35; 21.68]		0.340
Feel isolated from others				
No (ref.)				
Yes	0.38	[0.01; 26.37]		0.654
Church or religious activities				
Rarely	0.90	[0.45; 1.81]		0.776
Often (ref.)				
Religious activities at home				
Rarely	0.62	[0.31; 1.27]		0.192
Often (ref.)				
Adverse childhood experience	1.12	[0.72; 1.74]		0.607
Left home in the past one month during COVID				
No (ref.)				
Yes	1.34	[0.45; 4.00]		0.596
How often do you feel lonely during COVID				
Rarely or never/Some of the time (ref.)				
Occasionally/All of the time	5.99***	[2.87; 12.48]		<0.001
Type of dwelling				
House (ref.)				
Apartment	2.55*	[1.24; 5.24]		0.011
Others	5.61*	[1.08; 29.03]		0.040
Living alone during the COVID-19 pandemic				
No (ref.)				
Yes	0.90	[0.35; 2.32]		0.829
Functional limitation scale				
No (ref.)				
Yes	2.69**	[1.43; 5.05]		0.002
COVID _ Health stressors				
No (ref.)				
Yes	3.72***	[2.12; 6.55]		<0.001
COVID _ Difficulties with accessing resources				
No (ref.)				
Yes	1.39	[0.67; 2.91]		0.375
COVID _ Family conflict				
No (ref.)				
Yes	3.30**	[1.47; 7.43]		0.004
COVID _ Other family Issues				
No (ref.)				
Yes	1.38	[0.75; 2.55]		0.298
COVID _ Health care				
No (ref.)				
Yes	1.91*	[1.04; 3.50]		0.037
COVID _ Medications				
No (ref.)				
Yes	1.04	[0.38; 2.86]		0.942
Likelihood ratio test statistic	139.57***			
Nagelkerke R square	0.342			

*p <0.05

**p <0.01

***p <0.001

Table 4 presents the estimated parameters derived from the relative risk model, focusing on the depression status of older adults with a history of depression prior to the pandemic, specifically during CLSA Autumn 2020. Individuals who experienced loneliness occasionally/ all of the time during the initial months of the COVID-19 pandemic exhibited an elevated risk of developing depression by Autumn 2020, with a relative risk (RR) of 1.30 (95% CI [1.03; 1.63], p = 0.013). Older adults with functional limitations had a higher likelihood of experiencing depression compared to their counterparts without such limitations, with an RR of 1.50 (95% CI [1.17; 1.90], p<0.001). Moreover, respondents who reported increased levels of family conflict demonstrated a heightened probability of developing depression, with an RR of 1.52 (95% CI [1.17; 1.97], p<0.001). Additionally, individuals who encountered other family-related issues such as separation from family members and/or increased caregiving responsibilities had a 1.45 times higher risk of depression (RR = 1.45, 95% CI [1.05; 1.99], p = 0.001) compared to those who did not face such issues.

**Table 4:** Relative risk regression results for depression among older adults with PUD with a history of depression (n = 451).

	Relative Risk	95%CI	p-value
Age	0.99	[0.98; 1.01]	0.338
Sex			
Male (ref.)	0.99	[0.79; 1.26]	0.962
Female			
Marital status			
Married/Common-law (ref.)	1.08	[0.77; 1.51]	0.608
Separated/Divorced/Widowed	0.93	[0.62; 1.40]	0.677
Single			
Immigrant status			
No (ref.)	1.23	[0.90; 1.70]	0.129
Yes			
Visible minority status			
No (ref.)			
Yes	0.86	[0.50; 1.48]	0.551
Education			
Less than secondary school	0.89	[0.57; 1.39]	0.557
Secondary and some post-secondary	0.99	[0.75; 1.31]	0.927
Post-secondary degree/diploma (ref.)			
Household income			
Less than $49,999 (ref.)			
$50,000–$99,999	1.25	[0.92; 1.70]	0.098
$100,000 or more	1.12	[0.76; 1.65]	0.539
Missing	1.43	[0.83; 2.46]	0.126
Dwelling ownership			
Rent (ref.)			
Own with mortgage	1.09	[0.76; 1.55]	0.555
Own without mortgage	1.16	[0.81; 1.65]	0.359
Missing	0.87	[0.35; 2.16]	0.750
Total saving			
Less than $50,000 (ref.)			
$50,000–$99,999	0.85	[0.60; 1.21]	0.291
$100,000 or more	0.79	[0.59; 1.06]	0.078
Missing	0.65	[0.39; 1.08]	0.064
Whether income satisfies needs			
No	1.15	[0.86; 1.54]	0.277
Yes (ref.)			
BMI			
Underweight or normal weight (ref.)			
Overweight	0.82	[0.62; 1.09]	0.132
Obese	0.79	[0.59; 1.04]	0.060
Chronic pain			
No (ref.)			
Yes	1.24	[0.98; 1.57]	0.058
Multimorbidity			
0 (ref.)			
1	0.98	[0.66; 1.45]	0.919
2	1.06	[0.73; 1.54]	0.740
3+	1.06	[0.75; 1.51]	0.719
Missing	0.98	[0.52; 1.84]	0.946
ACE	0.98	[0.86; 1.12]	0.768
Feel they lack companionship			
No (ref.)			
Yes	1.06	[0.75; 1.51]	0.666
Feel left out			
No (ref.)			
Yes	0.95	[0.60; 1.49]	0.770
Feel isolated from others			
No (ref.)			
Yes	1.36	[0.89; 2.10]	0.046
Church or religious activities			
Rarely	1.32	[0.97; 1.80]	0.055
Often (ref.)			
Religious activities at home			
Rarely	1.20	[0.93; 1.54]	0.114
Often (ref.)			
Left home in the past one month during COVID			
No (ref.)			
Yes	0.75	[0.52; 1.07]	0.054
How often do you feel lonely during COVID			
Rarely or never/Some of the time (ref.)			
Occasionally/All of the time	1.30*	[1.03; 1.63]	0.013
Type of dwelling			
House (ref.)			
Apartment	0.84	[0.62; 1.14]	0.192
Others	1.04	[0.66; 1.63]	0.845
Living along during the COVID-19 pandemic			
No (ref.)			
Yes	1.25	[0.89; 1.75]	0.142
Functional limitation scale			
No (ref.)			
Yes	1.50***	[1.17; 1.90]	<0.001
COVID _ Health stressors			
No (ref.)			
Yes	1.04	[0.84; 1.30]	0.688
COVID _ Difficulties with accessing resources			
No (ref.)			
Yes	0.95	[0.73; 1.25]	0.667
COVID _ Family conflict			
No (ref.)			
Yes	1.52***	[1.17; 1.97]	<0.001
COVID _ Other family Issues			
No (ref.)			
Yes	1.17	[0.92; 1.49]	0.155
COVID _ Health care			
No (ref.)			
Yes	1.02	[0.80; 1.30]	0.851
COVID _ Medications			
No (ref.)			
Yes	1.45**	[1.05; 1.99]	0.001

*p <0.05

**p <0.01

***p <0.001

## Discussion

This longitudinal study revealed that approximately 13% of older Canadians with PUD who had no previous history of depression developed depression for the first time during the COVID-19 pandemic. Among those with a lifetime history of depression, approximately 47% experienced a recurrence of depression symptoms during the pandemic. For comparison, sensitivity analyses were conducted to examine the incidence and recurrence of depression among older adults with PUD during the pre-pandemic period between the Baseline (2011–2015) and Follow-up 1 (2015–2018). Our results indicate that the incidence and recurrence of depression pre-pandemic wave were 7.5% and 37.9%, respectively, which was substantially lower than the incidence and recurrence of depression during the pandemic. The findings of this study emphasize the importance of supporting the mental health of older adults with PUD during the COVID-19 pandemic. There were several factors that were associated with incident and recurrent depression, highlighting subpopulations of older adults with PUD who may be more vulnerable to adverse mental health outcomes.

Older women with PUD were approximately twice as likely to develop incident depression as their male counterparts. The high vulnerability to depression among older women has been previously identified in studies both before (e.g., [27]) and during the pandemic (e.g., [6]). The higher risk of depression identified in the current study may be partly due to declines in social interaction during the pandemic [28]. Although older women tend to have higher levels of social support than older men, a review on sex differences in depression among older adults hypothesized that older women may be more vulnerable to the absence of social support than their male counterparts [29]. Similarly, a study by Carayanni and colleagues [30] found that social support factors, such as regularly engaging in outings and excursions, were protective against depression for older women, but not for older men. It is possible that disruptions to socializing due to extended lockdowns disproportionately harmed older women. This is supported by the emerging research that has found higher levels of loneliness among older women compared to older men during the pandemic [31].

Among older adults with PUD, experiencing loneliness during the first few months of the pandemic was associated with a six-fold risk for incident depression and a more than two-fold risk for recurrent depression. There is a well-established link between loneliness and depression in middle-aged and older adults [27, 32]. Prior to the COVID-19 pandemic, loneliness and social isolation had already been identified as a major public health concern compromising the physical and mental health of older adults [33], and emerging research suggests that levels of loneliness among older adults have increased during the pandemic [34]. Increases in loneliness and time spent isolated are particularly concerning for older adults with PUD, as social isolation is associated with PUD recurrence in older adults with depression [35], emphasizing the reciprocal relationship between physical and mental health outcomes in older adults with PUD.

Among those with no history of depression, individuals who had trouble financially meeting their basic needs before the pandemic had more than triple the risk of developing depression for the first time during the pandemic. The COVID-19 pandemic created unprecedented economic challenges for vulnerable groups, particularly for those who live at the intersection of different vulnerabilities, such as low-income older adults with chronic illness [36]. Although older adults often have sources of income that would not have been affected by the pandemic, such as pensions and social security, many low-income older adults maintain employment into later life to meet their financial needs. Older adults are frequently employed in industries that were disproportionately impacted by periods of lockdown, such as retail stores, which may have resulted in layoffs and reductions in scheduling [36].

Among those with PUD, functional limitations were associated with an approximately three-fold risk for both incident and recurrent depression, while chronic pain was associated with a 63% higher risk of recurrent depression only. Functional limitations and chronic pain have important implications for the mental health of older adults. There is a high comorbidity between chronic pain and depression [37]. Both conditions are highly intertwined, whereby both physical and psychological pain exacerbate the other condition [37]. These findings of the current study are consistent with other research on the effects of chronic pain on depression, which indicate that high levels of pain can lead to depressive symptoms, potentially due to pain’s impact on physical functioning and fatigue [38]. Declines in physical functioning that lead to increased dependency have also been found to predict depression [39]. A longitudinal analysis of community-dwelling and institutionalized older adults found that those with limitations in activities of daily living (ADLs) and instrumental activities of daily living (IADLs) had significantly higher depression scores than those with no limitations [39]. Functional limitations and chronic pain often disrupt usual role activities, which is associated with lower quality of life among those with PUD [15, 16].

Older adults with PUD who reported increases in family conflict during the pandemic, such as verbal or physical conflict and/or marital breakdown, were three times more likely to develop incident depression and four times more likely to develop recurrent depression. Emerging research indicates substantial deterioration in family functioning during the pandemic [40]. COVID-19 has severely impacted many domains of life that may increase stressors among families, such as employment instability and economic precarity. When combined with increased time spent together at home in conjunction with disruptions to coping mechanisms that may reduce familial conflict, such as social support from loved ones, it is unsurprising that there may have been increases in interpersonal conflict [41]. Previous research has identified interpersonal conflict as a risk factor for depression in older adulthood [42].

There were several other COVID-19-related factors that were associated with incident and recurrent depression among older adults with PUD. Health stressors, such as being ill or having a loved one become ill during the pandemic, and/or experiencing the death of a loved one were associated with double the risk of incident depression only. Additionally, experiencing disruptions to regular health care access during COVID-19 was also associated with approximately double the risk of incident depression only. Finally, experiencing disruptions to medication access during COVID-19 was associated with a five-fold risk of recurrent depression only. The identified association between pandemic-related health stressors and depression supports other research that has found a high comorbidity between early COVID-19 infection and/or infection of a loved one and depressive symptoms [43, 44]. Additionally, the finding that disruptions to healthcare access and medication access were associated with depression reinforces issues with healthcare equity during COVID-19. There are ongoing concerns that reductions in healthcare access and shifts to telemedicine will create difficulties for managing chronic illness, particularly for older adults who may have challenges adapting to changing patterns of care [45]. Other studies have identified numerous medication-related problems for people with chronic illness during the pandemic, such as difficulty accessing medication due to doctor office closures, medication shortages, and fear of COVID-19 exposure in pharmacies or doctor’s offices, ultimately causing individuals to neglect obtaining their required medication [46]. It is essential to understand and address these issues in healthcare equity to support the mental health of older adults with PUD beyond the pandemic.

### Implications for intervention

It is important to consider interventions that can be used to support the mental health of older adults with PUD who may be vulnerable to depressive symptoms due to the COVID-19 pandemic. Usual treatments for depression, such as cognitive behavioral therapy (CBT) may be a viable and beneficial intervention for those experiencing concurrent depression and PUD, as CBT has demonstrated effectiveness for reducing depressive symptoms in older adults [47], and improving self-efficacy among older adults with chronic pain [48].

Other psychosocial interventions that have been shown to be helpful for supporting the mental well-being of older adults include mindfulness-based therapies, meditation, and problem-solving therapy (PST) [49, 50]. PST has shown to be more effective among those with later-life depression than in younger populations, in addition to individuals with comorbid conditions [49, 51], which may indicate its applicability to co-occurring depression and PUD.

Interventions that foster social connection may be particularly helpful in supporting the mental health of older adults during and beyond the COVID-19 pandemic. A recent systematic review examining potential interventions to reduce social isolation and loneliness during COVID-19 highlighted effectiveness of interventions that support social facilitation, particularly among older adults living in various congregate settings, such as assisted living and nursing homes [50]. For older adults living outside of these facilities, community care is a viable option for reducing depression in later life. Community care, a model of integrated partnership between local residents and community groups, provides older adults with social support programs, which promote the feeling of belonging and aging-friendly spaces [52]. Programs like older adult-youth partnerships, community gardens, and groups for those of particular communities and demographics, may offer a proactive option for mitigating depression among community-dwelling older adults. Creative strategies such as the above may be of particular importance moving forward when considering that many older adults have faced extended social isolation due to lockdowns and stay-at-home orders. As with any intervention, an important thread adding to its effectiveness among those who may be isolated or depressed is the active and sustained involvement of patients in their own care [53]. Practitioners should be attuned to ways in which they can curate such care to the specific needs of each older adult, such as listening to patient preferences, providing culturally competent care, and adapting to an individual’s ability in cases of cognitive impairment [53].

### Limitations

The current study has several limitations. First, although the CLSA provides rich longitudinal data, the observational nature of the current study precludes assuming causation in the reported associations. Second, depression was defined using the CES-D-10, which is a self-reported measure. Although it is a frequently used valid and reliable measure, it is not equivalent to a psychiatric assessment. However, the CES-D-10 has been found to have a sensitivity of 92% when compared with diagnostic interviews [54]. Third, the generalizability of the study may have been compromised because the following groups were excluded from data collection at Baseline: residents in the Northwest Territories, the Yukon, and Nunavut, and individuals who were residing in long-term care homes at the time of the Baseline interviews. Furthermore, although CLSA participants who moved to long-term care after the Baseline interview were included in the current study, lack of data from Canadians who were living in the long-term care system during the Baseline wave of data collection means the vulnerable subset of older adults living in long-term care settings may be under-represented. Those living in long-term care may be more susceptible to adverse mental health outcomes due to the extensive lockdown measures adopted in most long-term care settings in Canada.

### Conclusion

Despite the aforementioned limitations, the current research uses a large, longitudinal dataset to offer valuable insight into the mental health of older Canadians with PUD during the COVID-19 pandemic. As access to vaccines bring a gradual return to normalcy, clinicians should be attuned to the possible long-term mental health repercussions of COVID-19 among those experiencing chronic illness. The findings of this study indicate that many older adults with PUD may be experiencing depressive symptoms during the COVID-19 pandemic, emphasizing the need for ongoing mental health support and targeted interventions for this vulnerable population.
